# Double advantage of parental education for child educational achievement: the role of parenting and child intelligence

**DOI:** 10.1093/eurpub/ckac044

**Published:** 2022-05-12

**Authors:** Nathalie Tamayo Martinez, Yllza Xerxa, James Law, Fadila Serdarevic, Pauline W Jansen, Henning Tiemeier

**Affiliations:** The Generation R Study Group, Erasmus University Medical Center, Rotterdam, The Netherlands; Department of Child and Adolescent Psychiatry, Erasmus University Medical Center, Rotterdam, The Netherlands; The Generation R Study Group, Erasmus University Medical Center, Rotterdam, The Netherlands; Department of Child and Adolescent Psychiatry, Erasmus University Medical Center, Rotterdam, The Netherlands; School of Education, Communication and Language Sciences, Newcastle University, Newcastle-Upon-Tyne, UK; The Generation R Study Group, Erasmus University Medical Center, Rotterdam, The Netherlands; Department of Child and Adolescent Psychiatry, Erasmus University Medical Center, Rotterdam, The Netherlands; Department of Child and Adolescent Psychiatry, Erasmus University Medical Center, Rotterdam, The Netherlands; Department of Psychology, Education and Child Studies, Erasmus University Rotterdam, Rotterdam, The Netherlands; Department of Child and Adolescent Psychiatry, Erasmus University Medical Center, Rotterdam, The Netherlands; Department of Social and Behavioral Sciences, Harvard TH Chan School of Public Health, Boston, MA, USA

## Abstract

**Background:**

Parental education is one of the best predictors of child school achievement. Higher parental education is not only associated with higher child intelligence, but children from highly educated parents also perform better in school due to other family related factors. This study evaluates the relation between parental education, child non-verbal intelligence and parenting practices with child school achievement.

**Methods:**

Longitudinal data from a large population-based, multi-ethnic cohort of children in the Netherlands (63% Dutch origin) followed from birth to age 13 years (3547 children; 52.3% girls) were analyzed. School achievement was measured at the end of primary school (12 years of age) with a national Dutch academic test score. Parental education was assessed at age 3 years. The non-verbal intelligence of the child was measured at age 6 years and a full intelligence was measured at age 13 years. Maternal and paternal family routines, harsh parenting and corporal punishment were assessed in early and mid-childhood. Mediation analysis was performed with the G-formula and Structural Equation Models.

**Results:**

Child intelligence partially mediated [B indirect effect =0.54 95% CI (0.46, 0.62) *P* < 0.001] the association between parental education and child school achievement. Independent of intelligence, family routines [B indirect effect =0.04 95% CI (0.01, 0.07) *P* < 0.01], but not harsh parenting mediated this association.

**Conclusions:**

Higher parental education was associated with better school achievement through two independent mechanisms, through higher intelligence of the child and parenting practices.

## Introduction

Parental education is a consistent predictor of offspring school achievement and also of academic attainment, physical health, mental health and cognitive abilities.^1^^,^^2^ School achievement is an important developmental outcome and predicts final academic attainment, socioeconomic status^3^ and health throughout life.^4^

Parental education is associated with school achievement by mechanisms related to child intelligence^2^ and independent mechanisms. Parents with a higher intelligence generally have intelligent children,^5^ due to a high heritability of intelligence, which is around 20% in childhood, but increases to about 70% in adolescence and 80% in adulthood.^6^ Additionally, the family environment has lasting consequences on a child’s development; adopted children have a higher intelligence than non-adopted biological siblings and peers.^7^

Parents with a high education are able to provide social and material resources promoting a higher offspring school achievement. Higher educated parents tend to live in higher quality neighbourhoods,^8^ are more likely to provide cognitive enriched environments^9^ and tend to express expectations that children complete higher education.^10^ Parental education is related to parenting practices, such as family routines.^11^ Family routines refer to the stability in day-to-day activities, such as regular mealtimes, bedtimes and shared family activities,^12^ as well as regular individual activities like homework time. Family routines provide a sense of belonging and a predictable structure in the child’s life. Thereby, routines provide a stable emotional climate fostering healthy development.^13^ Family routines are associated with less anxiety, less depressive symptoms^14^ and higher intelligence.^13^ However, the association of family routines with school achievement has been studied less.

In contrast to family routines, harsh discipline, which includes the use of harsh verbal discipline or (mild) physical punishment,^15^ tends to occur more amongst parents with a lower education^15^ and can precede behavioural problems in children. Maltreatment is highly prevalent in high-income countries, with around 16% of children per year experiencing severe parental violence.^16^ Less serious forms of maltreatment like harsh parenting are even more common,^17^ and disturbingly, have also been associated with biological effects.^18^ It is important to evaluate mechanisms underlying harsh parenting to design strategies to prevent this parenting practice and its consequences.

Child characteristics may affect the parenting processes, yet few studies examined such a reverse association. Most of these studies focussed on child temperament, with children with a difficult temperament being more likely to receive negative parenting practices.^19^ Child intelligence is a critical characteristic that influences development and adult life outcomes.^20^ Moreover, a high intelligence is associated with ‘openness to experience’, which includes facets like active imagination, adventurousness, independence and intellectual curiosity.^21^ We hypothesize that intelligence can modify parenting practice. For instance, parenting children with low and high intelligence might pose different challenges due to the direct parental perception or due to child differences in personality and other mental health aspects.

In this study, we investigated mechanisms that differentially underlie the association of parental education with children’s school achievement. We focus on parenting practices and child intelligence, and as these concepts may fluctuate over time, repeated assessments are necessary.^22^ First, we studied parenting practices in early and mid-childhood (i.e. family routines, harsh parenting and corporal punishment) in the association of parental education and child school achievement, independently of child intelligence. Second, we quantified the independent mediating effect of repeatedly assessed child intelligence in the relation of parental education and child school achievement. Importantly, we accounted for reciprocal confounding, i.e. parenting practices or intelligence may each confound the mediated pathway.^23^ Third, we evaluated whether parenting and child intelligence jointly, i.e. in temporal sequence, mediate the association of parental education with child school achievement.

## Methods

### Participants

Participants were drawn from the Generation R Study, a population-based birth cohort that enrolled 9778 women in Rotterdam with an expected delivery date between April 2002 and January 2006. The study has been described elsewhere.^24^ This study has been approved by the Medical Ethical Committee of the Erasmus Medical Centre, and written informed consent was obtained from all participating parents. The eligible population for this study consisted of the 7393 children who participated in the school period. The study sample included only children with information on school achievement and parental education (*n* = 3490) (see flow chart, Supplementary figure S1). Additional information on the study sample, the assessment of school achievement and the early childhood parenting practices can be found in the Supplementary material.

### Maternal and paternal education

Information on their educational attainment was provided by mother and father during pregnancy, and at child ages 3 and 5 years. Education was scored as: primary education not completed; primary education completed; up to 3 years of secondary school; intermediate vocational training; higher vocational training or university degree. For the present analysis, we used the 3-year assessment.

### Offspring school achievement

The school achievement of the child was based on a test created by the Central Institute for Test Development (Dutch: Centraal Instituut voor Test Ontwikkeling, CITO), the CITO score. In the Netherlands, it is compulsory to administer an academic test in the final grade of primary school to guide the choice for secondary education (i.e. pre-vocational, higher general or pre-university level secondary education). Of the different tests, the CITO is the most frequently used. The test evaluates school achievement when children are 11–12 years old, by assessing language (e.g. in which sentence is a word spelled incorrectly? ‘This is the *eightst* long jumper’.) and mathematics skills (e.g. 7.7 + 3.07 = 10.14; 10.77; 10.71; or 11.40). The standardized test score ranges between 500 and 550, with higher scores pointing at a higher school achievement.

### Offspring intelligence

We measured children’s intelligence at two time points. A non-verbal IQ was determined when children were 6 years using the Dutch Snijders-Oomen non-verbal intelligence test.^25^^,^^26^ Mosaics and Categories are the subsets of the non-verbal IQ test used in Generation R. We chose a validated Dutch instrument and specifically investigated non-verbal IQ at this age, because our sample is multi-ethnic, and bilingualism is common; a valid assessment of verbal IQ before school age was not feasible. At age 13, four subtests of the Wechsler Intelligence Scale for Children-V were assessed, including Vocabulary, Matrix Reasoning, Digit Span and Coding scales.^27^ For this study, we assume that the IQ in middle childhood at 10–12 years is the same as the IQ assessed at 13 years.

### Parenting practices

We selected maternal and paternal parenting measures from different childhood periods. Early Childhood: family routines were reflected in a composite score derived from seven items about domains of family regularity reported by mothers when children were between 2 and 4 years old as described previously.^28^

Harsh discipline was assessed with a Dutch adapted version of the Parent–Child Conflict Tactics Scale,^15^ which mothers and fathers completed at child age 3 years. The harsh discipline scale is a self-report measure consisting of six items (e.g. ‘I shouted or screamed angrily at him/her’). Parents rated their use of this discipline practice in the last 2 weeks on a six-point scale.

Middle Childhood: corporal punishment was assessed with two questions from the Alabama Parenting Questionnaire.^29^ The mothers completed this questionnaire when the children were 8 years old, e.g. ‘Do you slap your child when he/she does something wrong?’. Mothers were asked how often this discipline type is used in the house, on a 5-point scale. The internal consistency (Cronbach’s alpha) was 0.67.

Family routines were measured with a subscale from the Stability of Activities in the Family Environment (SAFE) questionnaire.^30^ Both mothers and fathers completed this measure when children were 9 years. The scale is a validated self-report measure consisting of six questions (e.g. ‘How regular is your child’s homework routine after school’). Parents rated the regularity of the activities in the family during the last 6 months on a 4-point scale. In this study, the internal consistencies (Cronbach’s alpha) were 0.63 for the mother rated scale and 0.65 for the father rated scale.

### Covariates

The following possibly confounding factors were included. Maternal age was assessed at enrolment. Maternal national origin was categorized as Dutch, other Western and non-Western, based on her parents’ country of birth. A parent was categorized as Dutch origin if both her/his parents were born in the Netherlands, the Western category was created if either reported European or American Western origin and the non-Western category included Surinamese, Dutch Antillean, Turkish and all African descent amongst other origins. Mother’s IQ was estimated when the child age 6 years, with the set I from the Raven’s Advanced Progressive Matrices Test.

### Statistical modelling

We examined the correlation between the different parenting practices with the Spearman’s correlation coefficient. We evaluated the explained variance of parental education, child IQ and parenting practices in the association with school achievement.

**Figure 1:**
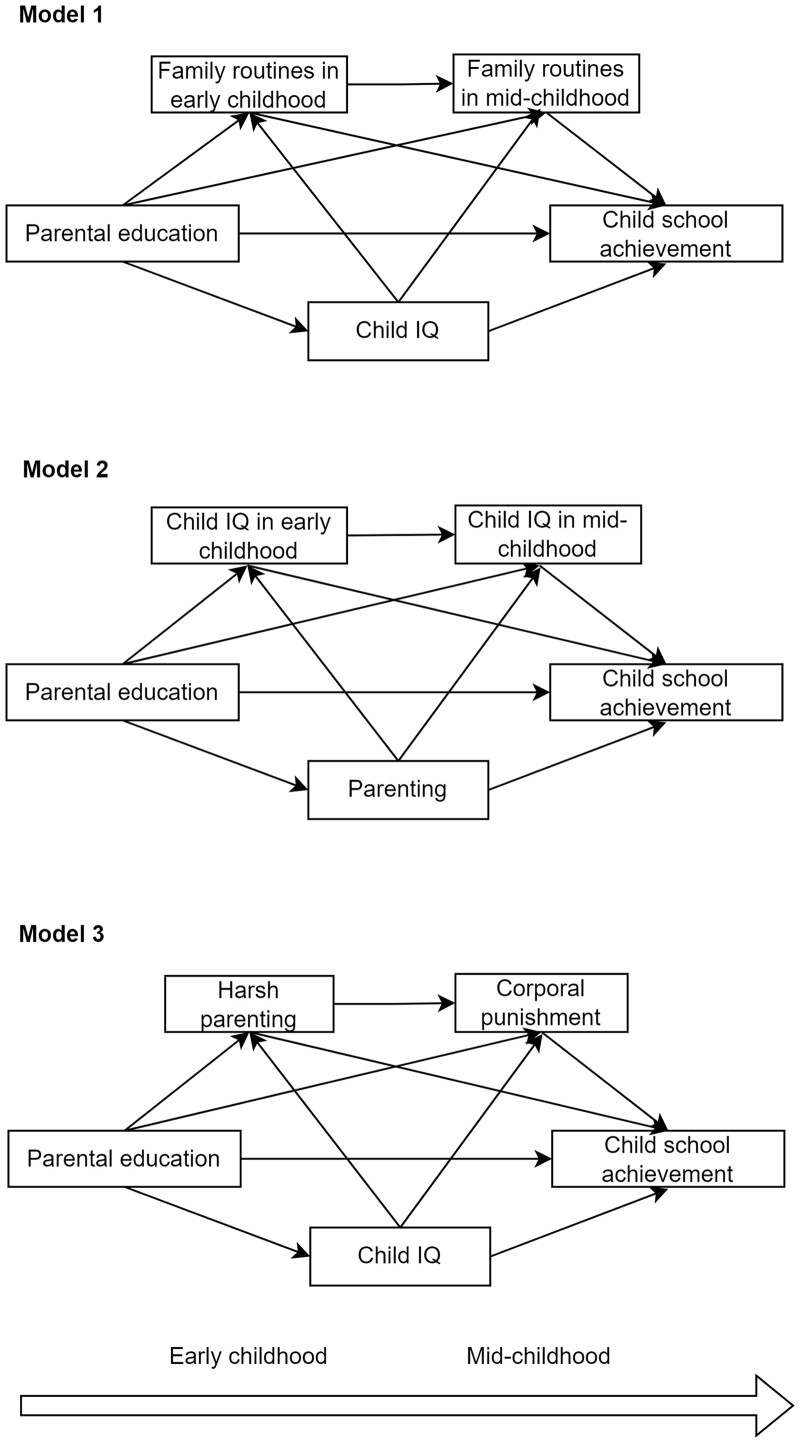
Outline of mediation models. Mediation models of parental education and child school achievement. The output is presented in table 2. In Model 1, we test the mediated effect of family routines independently of child IQ. In Model 2, we test the mediated effect of child IQ independently of parenting. In Model 3, we test the mediated effect of harsh parenting and corporal punishment independently of child IQ

To evaluate the first and second aim, we performed three mediation analyses using the G-formula from the CMAverse package in R. Specifically, we assessed the association between parental education and child school achievement and the role of the following three different mediators: (i) family routines in early and mid-childhood (see figure 1, Model 1); (ii) child intelligence in early and mid-childhood (see figure 1, Model 2); and (iii) harsh parenting in early childhood and corporal punishment in mid-childhood (see figure 1, Model 3). We evaluated the mediation paths in separate models to be able to account for confounding by parenting in the model of intelligence and by child intelligence in the model of parenting. For example, child characteristics like intelligence can elicit parenting styles^23^ and confound the mediation effects.^31^ The *P*-values were corrected for multiple testing with Benjamini and Yekutieli control that takes into account the dependency between measurements.^32^

To evaluate the third aim, how parenting practices and intelligence jointly mediate the impact of parental education on child school achievement, four Structural Equation Models were fitted with specific paths, each combining assessments in early and mid-childhood: (i) early childhood intelligence and mid-childhood corporal punishment (see Supplementary figure S2, Model 4); (ii) early childhood intelligence and mid-childhood family routines (see Supplementary figure S2, Model 5); (iii) early childhood harsh parenting and mid-childhood intelligence (see Supplementary figure S2, Model 6); and (iv) early childhood family routines and mid-childhood intelligence (see Supplementary figure S2, Model 7). These models all test sequential mediation, i.e. a combined mechanistic pathway. Standardized coefficients are presented.

Maternal and paternal education and the maternal and paternal scores of each parenting construct were combined with the mixed model approach^33^ using the Linear and Non-linear Mixed Effects Models (‘nlme’) package. This approach yielded combined latent parental variables, which were used in all analysis. All the models were adjusted for age, national origin and IQ of the mother.

In secondary analyses, we estimated the independent mediated effect of early or mid-childhood family routines in the association of parental education with child school achievement. Likewise, we tested the independent association of only early or mid-childhood child IQ. Second, we evaluated the association between parenting practices and child IQ in the full sample. In this analysis, we included all children regardless if they had information on school achievement (*n* = 7393 children).

Missing values on the parenting practices, covariates and child IQ were imputed using chained equations with mice package^34^ in R 3.5.3. The results presented are the estimates averaged across 20 imputed data sets. Additionally, we filled in missing data on parental education at 3 years, carrying the information from the pregnancy assessment forward or 5-year assessment backwards. We did not impute the school achievement data. We performed an attrition analysis comparing the 3547 included children to the 3846 children with missing information on school achievement. The included children had a higher non-verbal IQ (mean =104.0, SD = 14.4) than children not included (mean = 98.2, SD = 15.1), see Supplementary table S1 for detailed analysis.

## Results

The characteristics of the population are shown in table 1. Of the participants, 47.7% were boys. The mean non-verbal IQ of the children was 104.0 (SD = 14.4) and of the mother 98.3 (SD = 14.8). The national origin of most mothers was Dutch, 63.0%. The correlations between the parenting practices are presented in Supplementary table S2. Family routines in early and mid-childhood were positively correlated (ranging from *ρ* = 0.32–0.40). Similarly, harsh discipline in early childhood and corporal punishment in mid-childhood were correlated (ranging from *ρ* = 0.36–0.49). Family routines in early and mid-childhood were negatively correlated with harsh discipline and corporal punishment (ranging from *ρ* = −0.59 to −0.80). Of the variance of child school achievement, 14% was explained by parental education, 34% by child intelligence and each parenting practice explained around 1% (Supplementary table S3).

**Table 1:** Characteristics of the study population

Characteristics of the child	
Gender, boy, %	47.7
Age at academic test (in years), mean (SD)	11.9 (.4)
School achievement test (score), mean (SD)	538.4 (9.4)
Child non-verbal IQ (score), mean (SD)	104.0 (14.4)
Child full IQ (score), mean (SD)	104.6 (12.9)
Characteristics of the parents	
Mother’s age at intake (in years), mean (SD)	31.4 (4.7)
Mother IQ (score), mean (SD)	98.3 (14.8)
Maternal education, %	
Secondary school only, <3 years	14.4
Secondary school only, more than 3 years and less	27.2
Higher vocational training	27.0
University degree	31.3
Paternal education, %	
Secondary school only, <3 years	16.3
Secondary school only, more than 3 years	24.3
Higher vocational training	22.0
University degree	37.3
National origin of the mother, %	
Dutch	63.0
Western	8.0
Non-Western	29.0
Parenting practices	
Family routines mother age 4, median (IQR)	0.1 (0.6)
Harsh discipline mother age 3, median (IQR)	2.0 (2)
Harsh discipline father age 3, median (IQR)	2.0 (3)
Corporal punishment mother age 8, median (IQR)	0.0 (1)
Regularity in routines mother age 9, mean (SD)	18.4 (3.2)
Regularity in routines father age 9, mean (SD)	17.8 (3.4)

*Note*: Total *N* = 3547. Numbers denote children included in one or more analyses.

IQR, inter quartile range; SD, standard deviation.

The output corresponding to the first and second aim depicted in figure 1 are presented in table 2. Child IQ explained 42% [B indirect effect =0.54 95% CI (0.46, 0.62) *P* < 0.001] of the association between parental education and child school achievement. Additionally, regularity in the routines in early and mid-childhood mediated 3% [B indirect effect =0.04 95% CI (0.01, 0.07) *P* < 0.01] of this association. We did not find a statistically significant mediated effect of corporal punishment [B indirect effect =0.01 95% CI (−0.01, 0.02) *P* = 0.25].

**Table 2:** Independent mediated effect of child IQ and parenting practices in early and mid-childhood in the association between parental education and child school achievement

	School achievement		
Model	*β*	95% CI	*P* ^a^
1. Family routines in early and mid-childhood			
Indirect effect	0.04	(0.01, 0.07)	<0.01
Direct effect	1.25	(1.10, 1.39)	<0.001
Total effect	1.29	(1.14, 1.44)	<0.001
2. Child IQ in early and mid-childhood			
Indirect effect	0.54	(0.46, 0.62)	<0.001
Direct effect	0.75	(0.62, 0.88)	<0.001
Total effect	1.29	(1.14, 1.44)	<0.001
3. Harsh parenting and corporal punishment in early and mid-childhood			
Indirect effect	0.01	(−0.01, 0.02)	0.24
Direct effect	1.28	(1.13, 1.43)	<0.001
Total effect	1.29	(1.14, 1.44)	<0.001

*Note*: *N* = 3490. The mediation model output corresponds to Models 1, 2 or 3 in figure 1. In Model 1, we test the mediated effect of family routines independently of child IQ. In Model 2, we test the mediated effect of child IQ independently of parenting. In Model 3, we test the mediated effect of harsh parenting and corporal punishment independently of child IQ.

Models adjusted for age at enrolment and national origin of the mother and maternal IQ.

Standardized coefficients are presented. A 1 SD higher parental education is associated with a 1.29 SD higher child school achievement score. Of this, 0.54 SD score (42%) are through child IQ, 0.04 SD (3%) are through family routines.

a *P*-values adjusted for multiple testing.

The results of analyses examining the third aim are presented in table 3. There was no mediated effect through the sequential paths of early child IQ and mid-childhood parenting practice or through the sequential paths of early parenting practice and mid-childhood child IQ.

**Table 3:** Sequential mediated effect of child IQ and parenting practices in mid-childhood in the association between parental education and child school achievement

	School achievement		
Model	*β*	95% CI	*P*
4. Child non-verbal IQ and corporal punishment			
Indirect effect	−0.0001	(−0.0005, 0.0003)	0.35
Direct effect	0.30	(0.26, 0.34)	<0.001
Total effect	0.30	(0.26, 0.34)	<0.001
5. Child non-verbal IQ and family routines in mid-childhood			
Indirect effect	0.0002	(−0.0002, 0.001)	0.75
Direct effect	0.31	(0.27, 0.35)	<0.001
Total effect	0.31	(0.27, 0.35)	<0.001
6. Harsh parenting and child IQ			
Indirect effect	0.001	(−0.001, 0.002)	0.29
Direct effect	0.21	(0.18, 0.25)	<0.001
Total effect	0.21	(0.18, 0.25)	<0.001
7. Family routines in early childhood and child IQ			
Indirect effect	0.001	(−0.002, 0.004)	0.45
Direct effect	0.21	(0.17, 0.24)	<0.001
Total effect	0.21	(0.17, 0.24)	<0.001

*Note*: *N* = 3490. The mediation model outputs correspond to Models 4, 5, 6 or 7 in Supplementary figure S2. In Models 4 and 5, we test how the mediating effects of early childhood IQ depend on mid-childhood parenting practices. In Models 6 and 7, we test how the mediating effect of early childhood parenting depends on mid-childhood IQ.

Models adjusted for age at enrolment and national origin of the mother and maternal IQ. Standardized coefficients are presented. A 1 SD higher parental education is associated with a 0.21 or 0.31 SD higher child school achievement score.

The secondary analysis (Supplementary table S4) showed that early childhood IQ mediated the association of parental education with school achievement independently of mid-childhood IQ, but early childhood family routines did not mediate the relation of parental education with child school achievement independently of mid-childhood family routines [B indirect effect =0.02 95% CI (−0.003, 0.04) *P* = 0.11]. Mid-childhood family routines and IQ mediated the association of parental education with child school achievement independently of early childhood. Next, we contrasted the association between parenting practices and the child’s full IQ in the dataset of children with school achievement score to that in the complete data set. Results were very similar: harsh parenting and corporal punishment were negatively associated with child IQ in both datasets [Bs ≥ −0.56 95% CI (−1.10, −0.02) *P* = 0.04] (Supplementary table S5) and family routines were not related to child IQ [Bs ≤ 0.89 95% CI (−0.02, 1.80) *P* = 0.05].

## Discussion

In this study, using data from a population-based birth cohort, we examined the association between parental education, children’s intelligence and parenting practices in early and mid-childhood, with children’s school achievement at age 12 years. Our study results are in line with the positive association of parental education with the child’s intelligence and better school achievements. Additionally, we highlight two main findings. First, the child’s intelligence partially mediates the association between parental education and the child’s school achievement. Second, parental education is also related to offspring school achievement through parenting practices. Family routines mediated the relation between parental education and the child’s school achievement.

The positive association between parental education and offspring school achievement has been reported in numerous studies in over 50 years of research, across cultures and countries with different levels of economic development.^1^ We found that parental education was related to better school achievement of the child due the relation with offspring intelligence independent of the family routines, harsh parenting or corporal punishment. The child’s intelligence mediated around 40% of the association of parental education and the child’s school achievement. These analyses were controlled for maternal intelligence to minimize the impact of intellectual endowment of the mother.^6^ The association between parental education and child intelligence reflects the role of environmental and genetic factors in the association of parental education with the child’s intelligence in line with prior literature.

In this study, higher educated parents were more likely to provide more family routines, these routines partially mediated the association with child school achievement independently of child intelligence. Family routines including tasks related to education and cognition might help children achieve in school.^9^ Different aspects of family routines are beneficial for the child development, the repetitive nature of the activities provide predictability in the child’s environment^35^ that may make them less prone to attention problems.^28^ Children master skills by repetitive learning,^36^ routines support them to acquire academic competencies. Additionally, shared family routines, like sitting together for meals, allow parents to monitor their children’s behaviour and promote the bond between family members, fostering a sense of belonging. Arguably, the mediated effect by parenting practices was not large, it was 3% of the total effect. Yet, this is an important finding as school achievement is a complex outcome with numerous determinants and it points to routines as possible avenues for public health interventions.

Contrary to our hypothesis, there was no mediation by harsh discipline and corporal punishment in the relation between parental education and the child school achievement. This may be a result of the relatively low frequency, possibly reflecting a low actual prevalence and/or underreporting of harsh parenting and corporal punishment. Corporal punishment may not be very frequent in the Netherlands,^37^ more so because of a ban of some of these practices, like beating. Harsh discipline and corporal punishment were, however, associated with lower intelligence in line with previous literature.^38^ Given the consistent association between intelligence and school achievement, harsh parenting and corporal punishment cannot be ruled out as possible mediators of the relation of parental education and offspring school achievement.

There was no joint mediated effect by early childhood child intelligence and mid-childhood family routines or corporal punishment in the association between parental education and child school achievement. Although, child intelligence may elicit more regularity in the routines or less corporal punishment,^23^ this is not the path to a better school achievement. Similarly, the mediated effect of child intelligence was not explained by family routines or harsh parenting in early childhood. Ferretti and Bub observed that family routines before age 2 years were associated with higher child intelligence, but not family routines at age 3 years.^13^ Cognitive stimulating activities are common in structured families^12^ but may facilitate cognitive development only if occurring early in life.^13^ In contrast, harsh discipline was associated with lower intelligence, but we did not observe that this linked parental education to child school achievement, which may reflect lack of statistical power. Finally, timing remains a complex factor in child school achievement, as we found a mediated effect of mid-childhood family routines independent of early childhood family routines and an independent mediated effect of early and mid-childhood intelligence.

These results should be viewed against the background of some limitations. First, we did not have a father report for two parenting practices measures as we found it more difficult to motivate fathers to complete frequently mailed questionnaires. Therefore, family routines in early childhood were only rated by the mother, as was corporal punishment in mid-childhood. Parenting practices are a broad construct encompassing multiple behaviours in a wide time span that are hard to assess comprehensively. For instance, parental involvement is also related to parental education and child school achievement.^39^ Second, school achievement data were only available in part of the sample. However, this selection was largely determined by the test selection of schools. Third, the study sample has a relatively high socioeconomic background, which prompts caution when generalizing the results. Finally, the internal consistency of the parenting practices was low, we argue this is due to the small number of items in the parenting scales and not necessarily indicates low homogeneity.

This study has notable strengths, including the prospective nature of the data collection and the multiple ages at which parenting practices were collected minimizes recall bias from parental reports. The large number of children and the multi-ethnic composition of the sample improve the generalizability of the results. Further, both IQ and school achievement were objectively obtained in standardized settings.

These results illustrate that higher maternal and paternal education are important indicators of inequality in child school achievement for two different reasons; firstly, through the relation with higher child intelligence, and secondly, through the relation with more beneficial parenting practices. Importantly, implementing more routines, including morning, mealtime and bedtime routines, homework and school activities and household responsibilities could improve the child’s school achievement and may be a potential mechanism to narrow the gap between the children’s school achievement of higher and lower educated parents and hence social inequality. This, however, remains speculative. To interpret our results as causal, there must be no additional confounding between the exposure, the mediators and the outcome. Because we cannot be certain that we addressed all confounders, only a trial where researchers randomize parenting interventions or emulated trials could provide definitive causal evidence. Also, we postulate that some aspects of routines are amenable to school and community interventions and can be addressed by educational institutions, e.g. with supervised homework routines at school. Interventions in parenting practices have been shown to have lasting consequences in other areas of child development.^40^

## Supplementary data

Supplementary data are available at *EURPUB* online.

## Funding

The general design of the Generation R Study is supported by the Erasmus Medical Center-Rotterdam, the Erasmus University Rotterdam, the Netherlands Organization for Health Research and Development, the Netherlands Organization for Scientific Research, and the Ministry of Health, Welfare and Sport, the Municipal Health Service Rotterdam area, and the Stichting Trombosedienst and Artsenlaboratorium Rijnmond. Henning Tiemeier was supported by a grant of the Netherlands Organization for Scientific Research (NWO/ZonMW grant 016.VICI.170.200), Consortium on Individual Development, funding from the European Union Seventh Framework Program (FP7/2007-2013): ACTION: Aggression in Children: (grant number 602768).

This article was developed as part of the work of the SEED Consortium. SEED stands for Social InEquality and its Effects on child Development: a study of birth cohorts in the UK, Germany and the Netherlands (Grant # 462-16-030) and is part of the Dynamics of inequality across the lifecourse Programme of the EU’s New Opportunities for Research Funding Agency Co-operation in Europe (NORFACE) initiative. The consortium members are: Manja Attig, Gwendolin Blossfeld, Marie-Christine Franken, Wei Huang, Pauline Jansen, Claudia Karwath, Lisanne Labuschagne, James Law (PI), Cristina McKean, Robert Rush, Nathalie Tamayo Martinez, Hans-Günther Roßbach, Marc van der Schroeff, Jutta von Maurice, Helen Wareham and Sabine Weinert.

*Conflicts of interest*: None declared.

Key points

- Parental education is an important determinant of offspring education and is source of inequality through two different mechanisms.
- Children intelligence mediated 40% of the association of parental education and children school achievement.
- Highly educated parents provide more regular routines at home and this regularity is associated to a higher school achievement.
- Promoting routines in the activities at home might be a way to decrease school achievement inequality.

## Supplementary Material

ckac044_Supplementary_Data
