# Supplementary material for: Double advantage of parental education for child educational achievement: the role of parenting and child intelligence
Source: Eur J Public Health. 2022 May 12;32(5):690–5. doi: 10.1093/eurpub/ckac044 (PMC9527951; doi:10.1093/eurpub/ckac044)
Supplement: ckac044_Supplementary_Data [file ckac044_supplementary_data.pdf]

Double advantage of parental education for child educational achievement: the role of parenting and child intelligence

Supplementary Material

Table of contents

Participants 2

Offspring School Achievement 2

Parenting Practices in Early Childhood..3

Supplementary Table 1. Attrition comparison between the included and excluded children from the analysis 4

Supplementary Table 2. Correlations among parenting practices 5

Supplementary Table 3. Association between each exposure and mediator and child school achievement. 6

Supplementary Table 4. Independent mediated effect parenting practices or child IQ in early or mid-childhood in the association between parental education and child school achievement.....................7

Supplementary Table 5. Association of parenting practices and children's IQ in the dataset of children with school achievement measure and in the dataset of all the children who participated in the school period. 8

Supplementary Figure 1. Flowchart of the study population 9

Supplementary Figure 2. Sequential mediated effect of child IQ and parenting practices in mid-childhood in the association between parental education and child school achievement...................10

References 11

Participants

The eligible population for the present study consisted of the 7,393 children who participated in the school period (see flow chart, Supplementary Figure 1). Information on school achievement was obtained in 3,547 children, either by accessing the national database (n = 2,655) or maternal report when there was no data linkage (n = 892). Reasons for missing data on school achievement were: no consent to link the child's data to the national database; the test was not implemented in the child's school; the linkage was not successful, or there was no mother report available. We included only children with information on school achievement and parental education. Of the 3,547 children, 3,490 children had data of parental education. Additional information on the study sample and the assessment parenting practices can be found in the Supplementary Material.

Offspring School Achievement

The school achievement of the child was based on a test created by the Central Institute for Test Development (Dutch: Centraal Instituut voor Test Ontwikkeling, CITO), the CITO score. In the Netherlands, it is compulsory to administer an academic test in the final grade of primary school to guide the choice for secondary education (i.e., pre-vocational, higher general or pre-university level secondary education). Of the different tests, the CITO is the most frequently used. The test evaluates school achievement when children are 11-12 years old, by assessing language (e.g. in which sentence is a word spelled incorrectly?: This is the *eightst* long jumper.) and mathematics skills (e.g. 7.7 + 3.07 = 10.14; *10.77*; 10.71; or 11.40). The standardized test score ranges between 500 and 550, with higher scores pointing at a higher school achievement. For 1,295 children, we had information on the school achievement score both by linkage through the national database and the mother report. The correlation between these two assessments was .97 and 79.5% of mothers reported the correct school achievement

score. This suggests that mothers reliably report their children's CITO score. Therefore, for children with a maternal report only, we used this score.

Parenting Practices in Early Childhood

Family routines were reflected in a composite score derived from seven items about domains of family regularity reported by mothers when children were between 2 and 4 years old as described previously (1). The measure included two items on bedtime routines (e.g. 'Do you have a set pattern or ritual with your child at bedtime?') at age 2 years. At age 4, the measure included two items on family meal locations (e.g. 'How often do you have breakfast/evening meal around the table together with your child/children?') and three items on meal frequency (i.e. 'how often does your child eat breakfast/lunch/evening meals?'). A Confirmatory Factor Analysis was employed to combine these items into one construct. Model fit was assessed using the comparative fit index and the Tucker–Lewis index (CFI and TLI) and, the root mean square error of approximation (RMSEA), with acceptable fit being for CFI and TLI ≥ 0.90 and for RMSEA ≤ 0.06 (2). The model fit was acceptable (CFI = 0.94; TLI = 0.89; RMSEA = 0.05) (1).

Harsh discipline was assessed with a Dutch adapted version of the Parent-Child Conflict Tactics Scale (3), which mothers and fathers completed at child age 3 years. The harsh discipline scale is a self-report measure consisting of six items (e.g., "I shouted or screamed angrily at him/her"). Parents rated their use of this discipline practice in the last two weeks on a six-point scale. In the current study, internal consistencies (Cronbach's alpha) were low, 0.63 for the mother rated scale and 0.57 for the father rated scale. Most likely, this reflects the small number of items in the scale and the low prevalence of this parenting practice. In a model using a latent variable of harsh parenting with the six items we have shown a good model fit in this cohort (3) (mothers CFI = 0.97, TLI = 0.97, RMSEA = 0.04 and fathers CFI = 0.97, TLI = 0.97, RMSEA = 0.04).

Supplementary Table 1. *Attrition comparison between the included and excluded children from the analysis.*

	Included participants	Excluded participants
Characteristics of the child		
Gender, boy, %	47.7	52.1
Age at academic test (in years), Mean (SD)	11.9 (.4)	-
School achievement test (score), Mean (SD)	538.4 (9.4)	-
Child IQ (score) , Mean (SD)	104.0 (14.4)	98.2(15.1)
Characteristics of the parents:		
Mother's age at intake (in years), Mean (SD)	31.4 (4.7)	29.8 (5.3)
Mother IQ (score), Mean (SD)	98.3 (14.8)	93.4 (15.4)
Maternal education, %		
Secondary school only, less than 3 years	14.4	28.9
Secondary school only, more than 3 years and less	27.2	32.2
Higher vocational training	27.0	19.9
University degree	31.3	19.0
Paternal education, %		
Secondary school only, less than 3 years	16.3	26.7
Secondary school only, more than 3 years	24.3	28.4
Higher vocational training	22.0	20.2
University degree	37.3	24.7
National origin of the mother, %		
Dutch	63.0	48.1
Western	8.0	8.1
Non Western	29.0	43.8
Parenting practices		
Family routines mother age 4, Median (IQR)	.1 (.6)	-.03 (.7)
Harsh discipline mother age 3, Median (IQR)	2 (2)	3 (2)
Harsh discipline father age 3, Median (IQR)	2 (3)	3 (2)
Corporal punishment mother age 8, Median (IQR)	0 (1)	0 (1)
Regularity in routines mother age 9, Mean (SD)	18.4 (3.2)	17.9 (3.5)
Regularity in routines father age 9, Mean (SD)	17.8 (3.4)	17.5 (3.7)

Note. Total N included participants = 3,547, total N excluded participants = 3,846.

SD= Standard deviation

IQR= Inter quartile range

Supplementary Table 2. *Correlations among parenting practices.*

	1	2	3	4	5	6	7	8
Early childhood								
1. Family routines mother rating	1	-0.68	-0.59	-0.69	-0.63	0.40	0.32	0.40
2. Harsh discipline mother rating		1	0.70	0.95	0.49	-0.72	-0.69	-0.76
3. Harsh discipline father rating			1	0.88	0.36	-0.66	-0.68	-0.72
4. Harsh discipline combined, mother and father rating				1	0.48	-0.76	-0.74	-0.80
Middle childhood								
5. Corporal punishment mother rating					1	-0.66	-0.64	-0.69
6. Family routines mother rating						1	0.74	0.94
7. Family routines father rating							1	0.92
8. Family routines combined, mother and father rating								1

Spearman’s correlation coefficients are reported.

Supplementary Table 3. *Association between each exposure and mediator and child school achievement.*

	School achievement			
	β	*95% CI*	*p*	*Explained variance*
Parental education	0.41	(0.38, 0.44)	<0.001	13.98%
Parenting				
Family routines mother rated at age 4	0.11	(0.07, 0.14)	<0.001	1.08%
Harsh discipline combined, mother and father rating	-0.05	(-0.09, -0.01)	0.01	0.16%
Corporal punishment mother rating	-0.10	(-0.14, -0.06)	<0.001	0.97%
Family routines combined, mother and father rating ate age 9	0.10	(0.06, 0.14)	<0.001	0.91%
Non-verbal IQ	0.56	(0.53, 0.59)	<0.001	34.13%
Full IQ	0.14	(0.11, 0.17)	<0.001	

N= 3,490. Parental education and each parenting practice output is from one model. Both intelligence test outputs are form one model. Models adjusted for child gender, national origin and age at school achievement assessment. Standardized coefficients are presented. Positive coefficients represent increase in school achievement score and negative coefficients represent decrease in school achievement score.

Supplementary Table 4. *Independent mediated effect parenting practices or child IQ in early or mid-childhood in the association between parental education and child school achievement.*

	School achievement		
	β	*95% CI*	*p*
8. Family routines in early childhood			
Indirect effect	0.02	(-0.003, 0.04)	0.11
Direct effect	1.27	(1.13, 1.42)	<0.001
Total effect	1.29	(1.14, 1.44)	<0.001
9. Child IQ in early childhood			
Indirect effect	0.07	(0.04, 0.09)	<0.001
Direct effect	1.22	(1.08, 1.37)	<0.001
Total effect	1.29	(1.14, 1.44)	<0.001
10. Family routines in mid-childhood			
Indirect effect	0.02	(0.001, 0.04)	0.04
Direct effect	1.27	(1.12, 1.42)	<0.001
Total effect	1.29	(1.14, 1.44)	<0.001
11. Child IQ in mid-childhood			
Indirect effect	0.48	(0.40, 0.55)	<0.001
Direct effect	0.81	(0.68, 0.94)	<0.001
Total effect	1.29	(1.14, 1.44)	<0.001

N= 3,490. In Model 8, we test the mediated effect of early childhood family routines independently of mid-childhood family routines. In Model 9, we test the mediated effect of child IQ in early childhood independently of child IQ in mid-childhood. In Model 10, we test the mediated effect of mid-childhood family routines independently of early childhood family routines. In Model 11, we test the mediated effect of Child IQ in mid-childhood independently of child IQ in early-childhood. Harsh parenting and corporal punishment independent mediated effects were not assessed as there was no significant mediated effect in the main analysis. Models were computed with the G-formula, adjusting for age at enrollment and national origin of the mother and maternal IQ. Models 8 and 9 are adjusted for mid-childhood family routines or child IQ and models 10 and 11 are adjusted for early childhood family routines or child IQ. Standardized coefficients are presented.

Supplementary Table 5. *Association of parenting practices and children's IQ in the dataset of children with school achievement measure and in the dataset of all the children who participated in the school period.*

	Child IQ in children with school achievement data			Child IQ in all children		
	β	*95% CI*	*p*	β	*95% CI*	*p*
Early childhood						
Family routines mother rated	0.48	(-0.78, 1.74)	0.45	0.89	(-0.02, 1.80)	0.05
Harsh discipline combined, mother and father rating	-0.56	(-1.10, -0.02)	0.04	-0.56	(-0.97, -0.15)	<0.01
Middle childhood						
Corporal punishment mother rating	-0.75	(-1.23, -0.27)	<0.01	-0.77	(-1.12, -0.41)	<0.001
Family routines combined, mother and father rating	0.21	(-0.07, 0.49)	0.14	0.15	(-0.04, 0.35)	0.13

N= 3,490 for the dataset of children with school achievement measure and 7,393 for the dataset of all the children who participated in the school period. Each output is from one model. Models adjusted for age at enrollment and national origin of the mother, maternal IQ and parental education. Positive coefficients represent increase in IQ score and negative coefficients represent decrease in IQ score.

Supplementary Figure 1. *Flowchart of the study population*

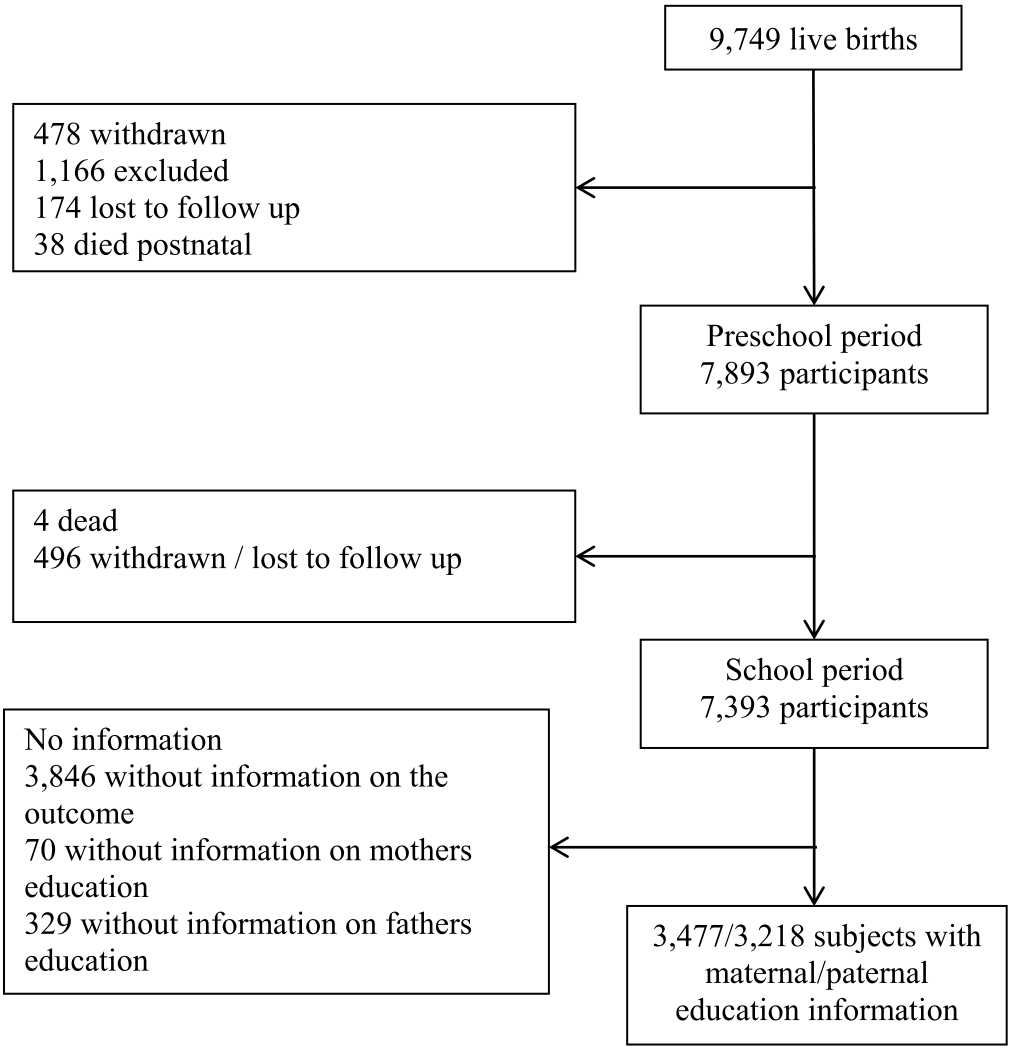

Supplementary Figure 2. *Sequential mediated effect of child IQ and parenting practices in mid-childhood in the association between parental education and child school achievement.*

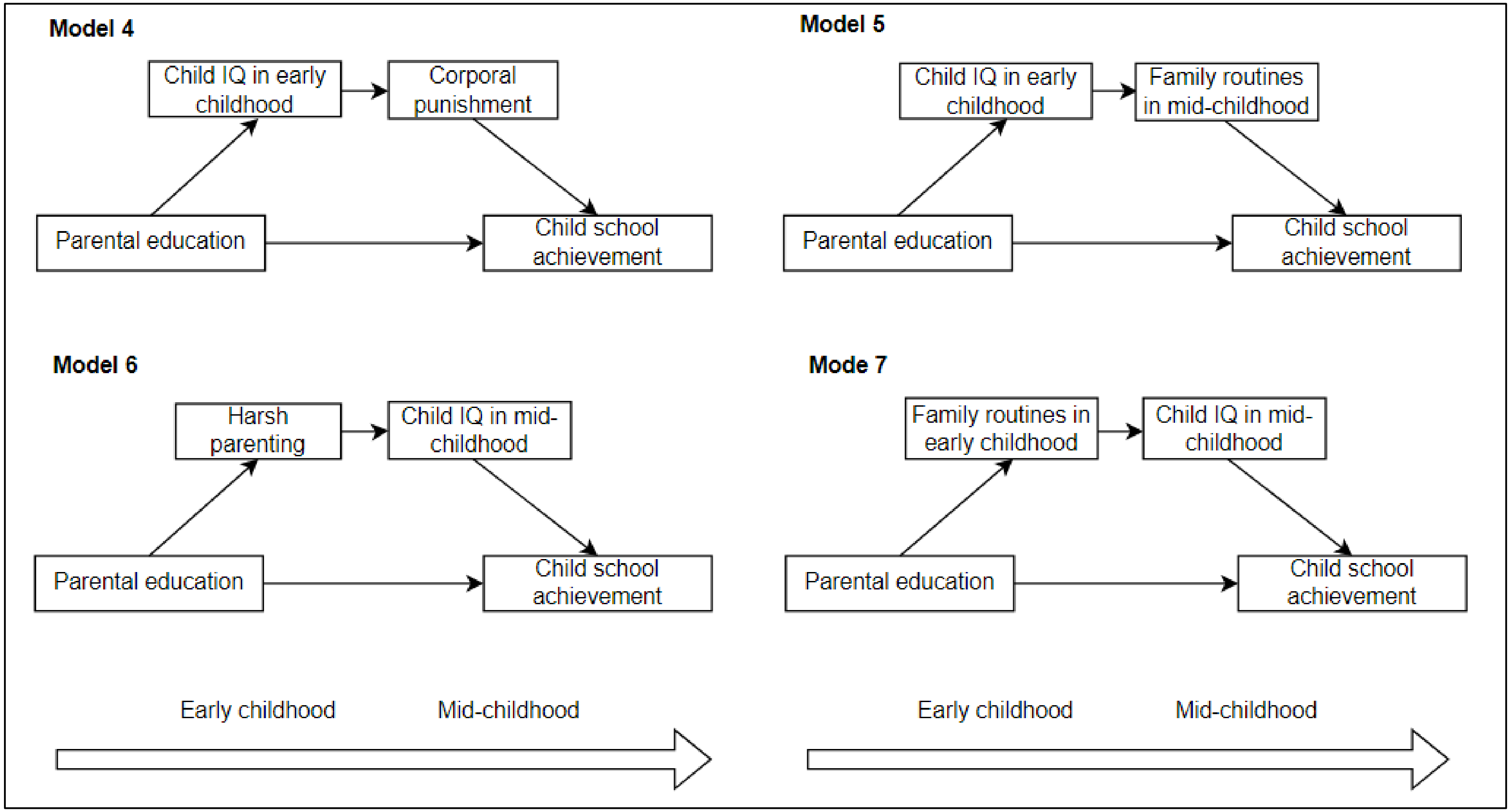

Mediation models of parental education and child school achievement. Outputs are presented in Table 3. In Models 4 and 5 the early childhood IQ mediated effect are dependent of mid childhood parenting practices. In Models 6 and 7, the early childhood parenting mediated effect are dependent of mid childhood IQ.

References

1. Rijlaarsdam J, Tiemeier H, Ringoot AP, Ivanova MY, Jaddoe VWV, Verhulst FC, et al. Early family regularity protects against later disruptive behavior. European Child & Adolescent Psychiatry. 2016;25(7):781-9.
2. Hu Lt, Bentler PM. Cutoff criteria for fit indexes in covariance structure analysis: Conventional criteria versus new alternatives. Structural Equation Modeling: A Multidisciplinary Journal. 1999;6(1):1-55.
3. Jansen P, Raat H, Mackenbach J, Hofman A, Jaddoe V, Bakermans-Kranenburg M, et al. Early Determinants of Maternal and Paternal Harsh Discipline: The Generation R Study. Family Relations. 2012;61:253-70.
